# Biochar Blended with Alkaline Mineral Can Better Inhibit Lead and Cadmium Uptake and Promote the Growth of Vegetables

**DOI:** 10.3390/plants13141934

**Published:** 2024-07-14

**Authors:** Lianxi Huang, Weisheng Chen, Lan Wei, Xiang Li, Yufen Huang, Qing Huang, Chuanping Liu, Zhongzhen Liu

**Affiliations:** 1 Laboratory of Plant Nutrition and Fertilizer in South Region, Laboratory of Nutrient Cycling and Farmland Conservation, Institute of Agricultural Resources and Environment, Guangdong Academy of Agricultural Sciences, Key, Ministry of Agriculture, Guangzhou 510640, China; hlx4@163.com (L.H.); 13538631591@163.com (W.C.); lixiang142213@163.com (X.L.); hyf0758@163.com (Y.H.); huangqingzh@gdaas.cn (Q.H.); 2 Institute of Eco-Environmental and Soil Sciences, Guangdong Academy of Sciences, Guangzhou 510650, China; cpliu@soil.gd.cn

**Keywords:** biochar, heavy metals, alkaline mineral, lead, cadmium, acidic soil

## Abstract

Three successive vegetable pot experiments were conducted to assess the effects on the long-term immobilization of heavy metals in soil and crop yield improvement after the addition of peanut shell biochar and an alkaline mineral to an acidic soil contaminated with lead and cadmium. Compared with the CK treatment, the change rates of biomass in the edible parts of the three types of vegetables treated with B0.3, B1, B3, B9, R0.2 and B1R0.2 were −15.43%~123.30%, 35.10%~269.09%, 40.77%~929.31%, −26.08%~711.99%, 44.14%~1067.12% and 53.09%~1139.06%, respectively. The cadmium contents in the edible parts of the three vegetables treated with these six additives reduced by 2.08%~13.21%, 9.56%~24.78%, 9.96%~35.61%, 41.96%~78.42%, −4.19%~57.07% and 12.43%~65.92%, respectively, while the lead contents in the edible parts reduced by −15.70%~59.47%, 6.55%~70.75%, 3.40%~80.10%, 55.26%~89.79%, 11.05%~70.15% and 50.35%~79.25%, respectively. Due to the increases in soil pH, soil cation-exchange capacity and soil organic carbon content, the accumulation of Cd and Pb in the vegetables was most notably reduced with a high dosage of 9% peanut shell biochar alone, followed by the addition of a low dosage of 1% peanut shell biochar blended with 0.2% alkaline mineral. Therefore, the addition of a low dosage of 1% peanut shell biochar blended with 0.2% alkaline mineral was the best additive in increasing the vegetable biomass, whereas the addition of 9% peanut shell biochar alone was the worst. Evidently, the addition of 0.2% alkaline mineral can significantly reduce the amount of peanut shell needed for passivating heavy metals in soil, while it also achieves the effect of increasing the vegetable yield.

## 1. Introduction

With the rapid development of the agricultural industry in recent decades, soil contamination by heavy metals has become a prominent problem in some regions of China [1]. Both the Ministry of Environmental Protection and the Ministry of Land and Resources reported that about 19% of the soil sampling sites were contaminated by either heavy metals or other pollutants in 2014. In particular, cadmium (Cd) and lead (Pb) are the most common heavy metals in arable land soil [2,3]. Therefore, it is an urgent matter to develop effective measures to remediate soils contaminated with heavy metals.

Various technologies including soil removal, pollutant dilution [4,5], bioremediation [6,7] and chemical solidification [8,9] have been developed to remediate soils polluted with heavy metals. Of these measures, chemical or physical immobilization are acute measures that focus on transforming heavy metals into less soluble and bioavailable forms through soil amendments and have been widely used to remediate soils contaminated by different heavy metals [10,11]. The exogenous addition of a material for solidifying heavy metals may cause secondary pollution to the soil and environmental resources; thus, the source and property of such a material are extremely important.

Of the numerous tested materials, including biochar, sepiolite and chitosan, biochar derived from the anoxic pyrolysis of plant biomass is considered an innovative material and has shown high potential as a soil conditioner [12,13,14]. A considerable number of laboratory and field tests have shown that biochar amendment can not only significantly reduce the mobility and bioavailability of heavy metals but also improve soil fertility and increase carbon sequestration [15,16,17,18]. However, the effects of biochar amendment depend on a bundle of factors, including the types of heavy metals, the polluted status, the soil conditions, and the nature and dosage of the biochar used [19,20,21,22,23,24]. Biochars with a high ash content and well-developed pores usually show better impacts on soil remediation [25,26]. Some heavy metals, such as Fe, Mn, Zn, Cd, Cu and Pb, are more strongly and readily adsorbed on the biochar surface [27]. Compared with alkaline soil, acidic soil commonly exhibits a positive response to biochar amendment for most heavy metals [28]. It is, thus, vital to use a proper type of biochar, depending on the characteristics of the biochar, the heavy metals present and the soil conditions.

Excessive biochar amendment may induce negative impacts on soil and pose a high cost as well [29,30]. Recent research studies have shown that the combination of biochar amendment with other measures may achieve additional benefits. For example, biochar amendment combined with the use of either a mineral or manure has much better impacts on nutrient use efficiency [31]. The combined amendment of biochar with alkaline materials, such as lime, fly ash and alkaline mineral, might result in the stronger immobilization of heavy metals and reduce the accumulation of heavy metals in plants [32,33]; the effects largely depend on not only the biochar used and its addition rate but also the heavy metals and soil conditions such as acidity. Therefore, it is meaningful and valuable to have a full understanding of the combination of biochar amendment with alkaline minerals since a large volume of alkaline industrial wastes is released in China and other countries.

In this study, peanut shell biochar was amended with a kind of alkaline industrial waste into Pb–Cd-contaminated acidic soil, and then the soil was used to successively grow three types of vegetables in pots. We hypothesized that the co-application of biochar with the alkaline residues might lead to the better immobilization of both Pb and Cd, compared with the use of each alone. The purposes of this study were to (1) determine the proper amendment rate of peanut shell biochar by evaluating the consecutive effects on the bioavailability of soil Pb and Cd and accumulation in plants; (2) investigate the interaction of peanut shell biochar with an industrial alkaline mineral; and (3) recommend the proper co-application of peanut shell biochar with industrial alkaline residues in acidic soil.

## 2. Materials and Methods

### 2.1. Soil, Biochar and Alkaline Mineral

Acidic red soil of the Oxisol type collected from a mining site (113°30′–114°02′ E, 24°56′–25°27′ N) in Guangdong Province of China was sieved through a 2 mm sieve, followed by air drying. Peanut shell biochar was used in this experiment because of its good absorption to Cd and Pb and easy access as a raw material [34]. The peanut shell was pyrolyzed in a biomass carbonization furnace under an oxygen-limited condition at 500 °C for 2 h and then cooled in a steel tank. After preparation, the peanut shell biochar was sieved through a 60–80-mesh sieve and stored in sealed plastic bags. The alkaline mineral used in this study was supplied by an alkali factory in Guangdong Province of China. The fundamental properties of the soil, biochar and alkaline mineral are shown in Table 1. The soil was heavily contaminated by both Pb and Cd, while the biochar and alkaline mineral had low contents of Pb and Cd.

### 2.2. Pot Experiment

The pot experiment involving the use of 7 kg of soil in a pot was carried out in a greenhouse. The air-dried and crushed soil was thoroughly mixed with a series of peanut shell biochar (0.3%, 1%, 3% and 9% in mass, labeled as B0.3, B1, B3 and B9, respectively), alkaline mineral (0.2% in mass, labeled as R0.2), and 1% peanut shell biochar plus 0.2% alkaline mineral (labeled as B1R0.2). The soil without addition was used as a control (CK). Each treatment was conducted in four pots as four replicates. After thoroughly mixing with 200 mg kg^−1^ KCl, 340 mg kg^−1^ (NH_4_)_2_HPO_4_ and 61.5 mg kg^−1^ CO (NH_2_)_2_, all soils were adjusted to have a 70% water-holding capacity and conditioned in a sealed tank at room temperature for 10 d. Crown daisy (*Chrysanthemum coronarium* L., CC) was first cultured in a glasshouse for 53 days, and then lettuce (*Lactuca sativa* L. var. *ramosa* Hort., LSr) was cultured for 37 days, followed by long-leaf lettuce (*Lactuca sativa* L., LS) for 78 days, after the harvest of crown daisy. Additional inorganic fertilizers, including 200 mg kg^−1^ KCl, 340 mg kg^−1^ (NH_4_)_2_HPO_4_ and 61.5 mg kg^−1^ CO (NH_2_)_2_, were added during the cultivation of lettuce and long-leaf lettuce.

At the end of cultivation, both the shoots and roots of the vegetable plants were harvested from each pot and measured for fresh mass. Following rinsing with tap water and then deionized water, they were oven-dried at 105 °C and then measured for the dry matter and Pb and Cd contents. At the same time, soil samples were collected from each pot. After being air-dried, the soil samples were determined for pH value, cation-exchange capacity (CEC), organic carbon (OC), total Pb, total Cd, available Pb and available Cd.

### 2.3. Assay

The pH values of the soil and biochar samples were determined using an acidometer (Mettle S210-K made by METTLER TOLEDO company, Columbus, OH, USA) at a 1:2.5 ratio of soil to deionized water and a 1:10 ratio of biochar to deionized water, respectively. OC was determined using the potassium dichromate–sulfuric acid oxidation method, and CEC was determined using the ammonium acetate exchange method. Available N, P and K were quantified using the alkalolytic diffusion method, sodium bicarbonate extraction–molybdenum antimony resistance colorimetric method and ammonium acetate extraction–flame photometry method, respectively. Available Cd and Pb were extracted with diethylenetriamine pentaacetic acid (DTPA) and then determined using an atomic absorption photometer fitted with a graphite furnace (PE AA600 made by PE company, New York, NY, USA). Total Pb and Cd in the soil samples were completely digested with hydrochloric acid, nitric acid, hydrofluoric acid and peracetic acid and then detected via atomic absorption spectrometry. Total Pb and Cd in the plant samples were completely digested with hydrochloric acid, nitric acid and perchloric acid and then determined via atomic absorption spectrometry.

### 2.4. Statistical Analysis

All data were evaluated as the means of four replicates and expressed on the basis of the oven-dried base. Significant differences among the treatments of each plant were analyzed with one-way ANOVA using the SAS.V9 software. The same lowercase letters shown in the figures indicate non-significant difference between the same experimental treatments, while different lowercase letters indicate significant difference (*p* < 0.05). In the tables showing the results of the correlation analysis, * and ** indicate significant correlation at the 0.05 and 0.01 levels (bilateral), respectively. Correlation coefficients of 0.8~1.0, 0.6~0.8, 0.4~0.6, 0.2~0.4 and 0.0~0.2 represent strong correlation, moderate correlation, weak correlation, very weak correlation and no correlation, respectively. A negative value indicates a negative correlation.

## 3. Results

### 3.1. The Impacts of Amendments with Both Peanut Shell Biochar and Alkaline Mineral on Soil Properties 

#### 3.1.1. Soil pH, OC and CEC

**Figure 1:**
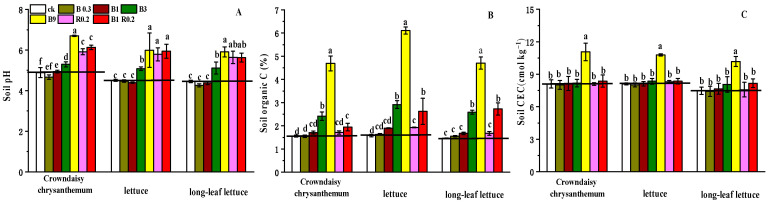
The values of pH (**A**), organic carbon (**B**) and CEC (**C**) in the soils amended with different additives and used for growing three types of vegetables. B0.3, B1, B3 B9, R0.2 and B1R0.2 represent 0.3% peanut shell biochar, 1% peanut shell biochar, 3% peanut shell biochar, 9% peanut shell biochar, 0.2% alkaline mineral and 1% peanut shell biochar plus 0.2% alkaline mineral in mass, respectively. The different lowercase letters above each bar indicate significant differences among the treatments for each vegetable plant.

The pH values of the control soil samples decreased gradually from an initial pH value of 4.73 to a pH value of 4.46 during the final cultivation with long-leaf lettuce, which might imply soil acidification during successive cultivation (Figure 1A). Adding less than 1% peanut shell biochar (B0.2 and B1) had little effect on soil pH value (*p* < 0.05). The soil pH values of the other treatments (B3, B9, R0.2 and B1R0.2) observably increased by 0.41–1.82 units. The increase in pH in acidic soil due to the application of additives was in the order of 9% peanut shell biochar alone, 0.2% alkaline mineral combined with 1% peanut shell biochar, 0.2% alkaline mineral alone, 3% peanut shell biochar alone and, finally, a low dosage of peanut shell biochar alone. 

The successive cultivation of the three types of vegetables did not significantly increase the soil organic carbon (SOC) content of the control treatment (Figure 1B). Adding less than 1% biochar and 0.2% alkaline mineral did not significantly change the SOC content. However, the SOC content increased by 25.00–285.40% with the addition of more than 3% peanut shell biochar and the mixture of 0.2% alkaline mineral plus 1% peanut shell biochar, which could be attributed to the added organic C from the peanut shell biochar (Table 1). 

The tested soil had a very low cation-exchange capacity (CEC) value (Table 1). Adding 9% peanut shell biochar (B9) largely enhanced the CEC by 9.23% after cultivating the three types of vegetables, while other amendments did not significantly change the soil CEC (Figure 1C). 

#### 3.1.2. Soil Available Cd and Pb

**Figure 2:**
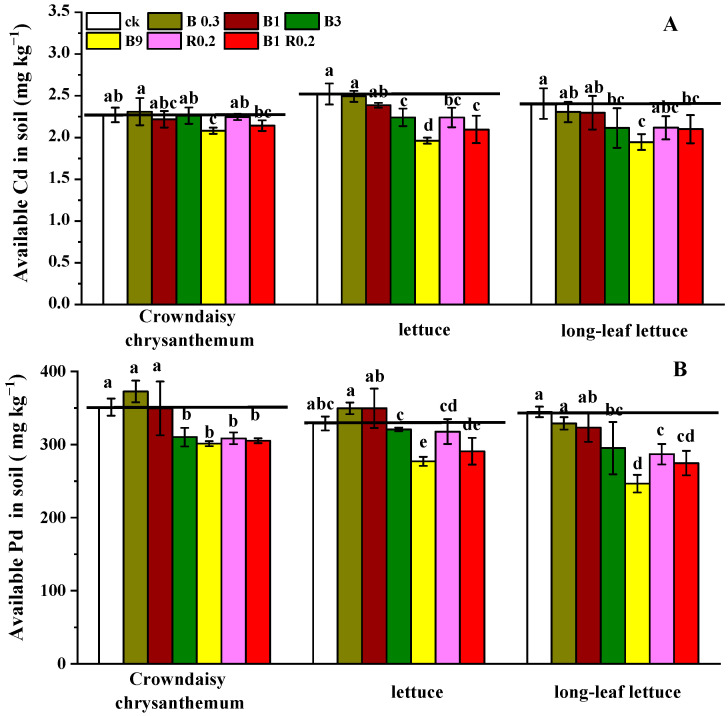
Available contents of Cd (**A**) and Pb (**B**) in the soils amended with different additives and used for growing three types of vegetables. B0.3, B1, B3 B9, R0.2 and B1R0.2 represent 0.3% peanut shell biochar, 1% peanut shell biochar, 3% peanut shell biochar, 9% peanut shell biochar, 0.2% alkaline mineral and 1% peanut shell biochar plus 0.2% alkaline mineral in mass, respectively. The different lowercase letters above each bar indicate significant differences among the treatments for each vegetable plant.

The tested soil was heavily polluted by both Cd and Pb (Table 1). Cultivating the three types of vegetables did not significantly change the soil-available Cd and Pb (Figure 2). Adding less than 1% peanut shell biochar induced few changes in either available Cd or Pb. However, other amendment treatments, such as B3, B9, R0.2 and B1R0.2, resulted in a detectable reduction by 11.98–19.07% and 14.35–28.47% in available Cd and Pb, respectively, after the successive cultivation of the three types of vegetables compared with the values of the CK treatment. Available Cd and Pb in the soil amended with both 1% biochar and 0.2% alkaline mineral (B1R0.2) were 1.70–16.80% lower than those of the soil amended with either 1% biochar (B1), 0.2% alkaline mineral (R0.2) or the treatment with a higher dosage of biochar (3%), showing the positive interaction between the biochar and alkaline mineral in reducing the bioavailability of both Cd and Pb. 

### 3.2. The Impacts of Amendments with Both Peanut Shell Biochar and Alkaline Mineral on Vegetable Plants

#### 3.2.1. Increase in Vegetable Yields

**Figure 3:**
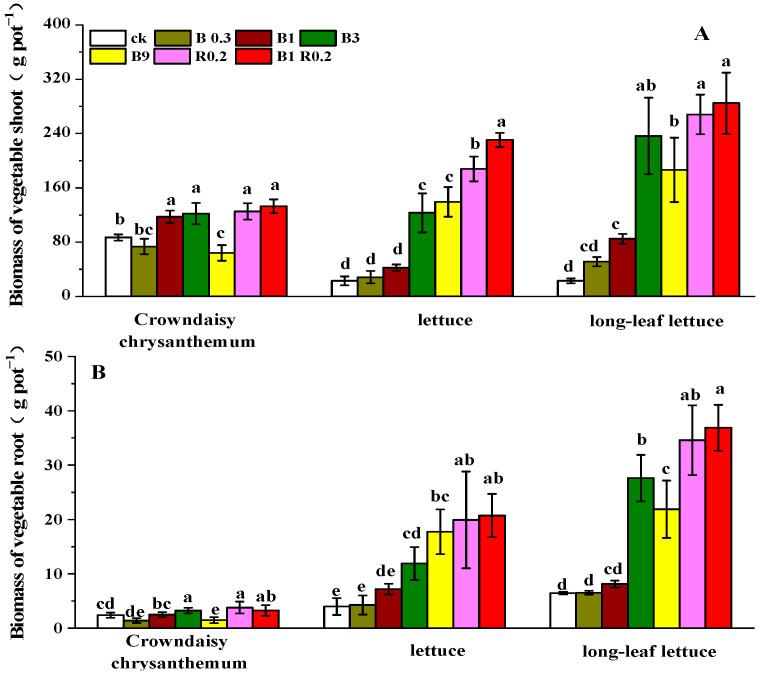
The fresh matter mass of the shoots (**A**) and roots (**B**) of crown daisy, lettuce and long-leaf lettuce grown in soils amended with different additives. B0.3, B1, B3 B9, R0.2 and B1R0.2 represent 0.3% peanut shell biochar, 1% peanut shell biochar, 3% peanut shell biochar, 9% peanut shell biochar, 0.2% alkaline mineral and 1% peanut shell biochar plus 0.2% alkaline mineral in mass, respectively. The different lowercase letters above each bar indicate significant differences among the treatments for each vegetable plant.

The control treatment (CK) had a very low fresh matter mass in either the shoots or roots. Adding 0.3% peanut shell biochar (B0.3) induced little change in the plants’ fresh matter (Figure 3A,B). However, the other amendments, B1, B3, B9, R0.2 and B1R0.2, resulted in a much higher fresh matter mass than the CK treatment. The biomass of the edible parts of crown daisy, lettuce and long-leaf lettuce treated with the six additives, including 0.3% peanut shell biochar, 1% peanut shell biochar, 3% peanut shell biochar, 9% peanut shell biochar, 0.2% alkaline mineral alone and 1% peanut shell biochar blended with 0.2% alkaline mineral, increased by −15.43%~123.30%, 35.10%~269.09%, 40.77%~929.31%, −26.08%~711.99%, 44.14%~1067.12% and 53.09%~1139.06%, respectively. Both lettuce plants showed better responses to the amendments than crown daisy, which even responded negatively to the amendment with 9% biochar. 

#### 3.2.2. Reduction in Cd Accumulation in Vegetable Tissues

**Figure 4:**
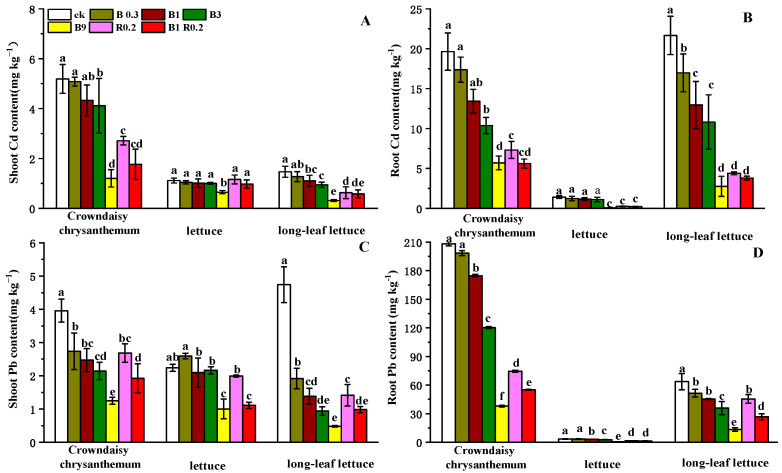
The Cd contents in the shoots (**A**) and roots (**B**) and the Pb contents in the shoots (**C**) and roots (**D**) of the three vegetables planted in soils amended with different additives. B0.3, B1, B3 B9, R0.2 and B1R0.2 represent 0.3% peanut shell biochar, 1% peanut shell biochar, 3% peanut shell biochar, 9% peanut shell biochar, 0.2% alkaline mineral and 1% peanut shell biochar plus 0.2% alkaline mineral in mass, respectively. The different lowercase letters above each bar indicate significant differences among the treatments for each vegetable plant.

The edible shoots of all three vegetables contained a non-negligible amount of Cd and Pb, ranging from 0.31 mg kg^−1^ to 5.19 mg kg^−1^ and 0.48 mg kg^−1^ to 4.74 mg kg^−1^, respectively (Figure 4A,C). The contents of Cd and Pb in the shoots of crown daisy were higher than those in the shoots of the other two lettuce varieties, with the highest treatment reaching values that were 3.88 and 1.58 times higher, respectively, indicating the stronger accumulation capacity of crown daisy for both heavy metals. The shoots of all three vegetables treated with B0.3, B1, B3, B9, R0.2 and B1R0.2 had a lower content of Cd of 2.08–13.21%, 9.56–24.78%, 9.96–35.61, 41.96–78.42%, –4.19–57.07% and 12.43–65.92%, respectively, as well as a lower content of Pb of 1.22–59.47%, 6.55–70.75%, 3.40–80.10%, 55.26–89.79%, 11.05–70.15%% and 50.35–79.25%, respectively, compared with the CK treatment.

### 3.3. The Effects of Amendments with Peanut Shell Biochar and Alkaline Mineral on the Absorption and Transport of Heavy Metals in Vegetables

**Figure 5:**
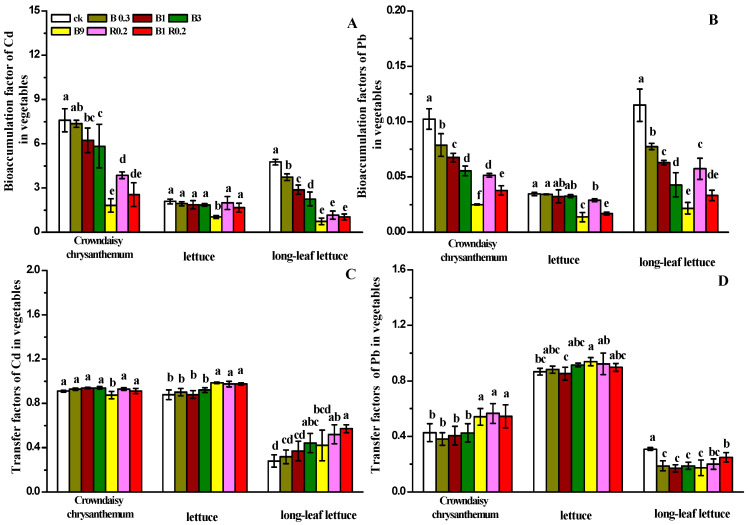
BFs of Cd (**A**) and Pb (**B**) and TFs of Cd (**C**) and Pb (**D**) in the three vegetables planted in soils amended with different additives. B0.3, B1, B3 B9, R0.2 and B1R0.2 represent 0.3% peanut shell biochar, 1% peanut shell biochar, 3% peanut shell biochar, 9% peanut shell biochar, 0.2% alkaline mineral and 1% peanut shell biochar plus 0.2% alkaline mineral in mass, respectively. The different lowercase letters above each bar indicate significant differences among the treatments for each vegetable plant.

The bioaccumulation factors (BFs) and transfer factors (TFs) of Cd and Pb shown in Figure 5 were used to evaluate the capacity of the three vegetables for Cd/Pb accumulation and Cd/Pb transfer from roots to shoots, as well as to explore the changes in Cd/Pb BFs and TFs due to the application of different dosages of peanut shell biochar and alkaline mineral additives. The changes in Cd BFs, shown in Figure 5A, and Pb BFs, shown in Figure 5B, due to the application of different dosages of peanut shell biochar and alkaline mineral additives are consistent with the changes in the Cd and Pb contents shown in Figure 4 because the BFs of Cd and Pb are mainly affected by their accumulation in the shoots and roots of the three vegetables. The treatments with B9 and B1R0.2 resulted in the lowest Cd BFs and Pb BFs in the three vegetables. A high dosage of peanut shell biochar and alkaline mineral additives significantly increased the Cd TFs of long-leaf lettuce and Pb TFs of crown daisy.

### 3.4. Analysis of the Main Factors Affecting the Content of Available Cd/Pb in Soil and Total Cd/Pb in Vegetables

The correlation analysis between available heavy metal contents in soil and general chemical characteristics, including soil pH, OC and CEC values, showed that the available Pb/Cd contents in soil were significantly negatively correlated with soil pH, OC and CEC (Table 2). The increase in soil pH value was the main reason for the decrease in soil Pb and Cd availability among the three factors. The Cd/Pb contents in the three vegetables were significantly negative correlated with the soil pH, OC and CEC values (Table 2) but significantly positive correlated with the available Cd/Pb contents in soil (Table 3). 

## 4. Discussion

The growth of crown daisy was significantly promoted by the application of a low dosage of 1% peanut shell biochar and a medium dosage of 3% peanut shell biochar, but it was suppressed by high dosages of 0.3% and 9% peanut shell biochar. The growth of lettuce and long-leaf lettuce was enhanced with an increased dosage of peanut shell biochar due to their higher tolerance for this additive. An analysis of the relationship between soil characteristics and vegetable growth showed that the biomass of lettuce and long-leaf lettuce increased with increases in soil pH, OC and CEC, but soil pH, CEC and OC had non-significant or negative correlations with the growth of chrysanthemum coronarium. These results suggest that the growth of vegetables is not only related to external factors such as soil physical and chemical properties but also affected by the growth characteristics of the vegetables themselves. The existing data had shown that proper biochar amendment drastically enhanced plants’ fresh matter through improving the soil physicochemical properties or soil fertility [35]. In particular, the positive effects of alkalic materials such as biochar and alkaline mineral were more pronounced in soils with low fertility and acidity [36]. The soil tested in this study was very acidic and exhibited satisfactory response to the addition of either peanut shell biochar or alkaline mineral. Moreover, the growth of crown daisy, lettuce and long-leaf lettuce was better promoted by the mixture of 0.2% alkaline mineral and a low dosage of 1% peanut shell biochar than by 0.2% alkaline mineral or any other dosages of peanut shell biochar alone. The treatment with a mixture of 0.2% alkaline mineral and 1% peanut shell biochar resulted in the highest fresh matter mass for all three vegetables, implying a significant and positive interaction between the peanut shell biochar and alkaline mineral. The best treatment in reducing the accumulation of Cd and Pb in crown daisy, lettuce and long-leaf lettuce was a high dosage of 9% peanut shell biochar, followed by 0.2% of alkaline mineral mixed with a low dosage of 1% peanut shell biochar. The reduction in the accumulation of Cd and Pb in the three types of vegetables by the aforementioned two treatments was significantly higher than by the treatments with 0.2% alkaline mineral or other dosages of peanut shell biochar alone. These results clearly indicated the inhibition of heavy metal uptake by the vegetables since the amendments with biochar at a higher rate could result in much lower contents of both Cd and Pb. The shoots of the three vegetable plants treated with the mixture of 0.2% alkaline mineral and a low dosage of 1% peanut shell biochar had lower contents of Cd and Pb of 3.17–59.17% and 22.11–46.87%, respectively, compared with the plants treated with either 1% peanut shell biochar, 3% peanut shell biochar or 0.2% alkaline mineral, which showed the significant and positive interaction between the added biochar and alkaline mineral in reducing the accumulation of these two heavy metals. There was no doubt that a high dosage of peanut shell biochar was the most optimal additive to immobilize soil Cd and Pb. However, the higher the dosage of peanut shell biochar, the higher the cost of its application in immobilizing soil Cd and Pb, and the more serious the inhibition effect on crop growth. The effect on Cd and Pb immobilization using the mixture of 0.2% alkaline mineral and a low dosage of 1% peanut shell biochar was only second to the treatment with a high dosage of 9% peanut shell biochar, which had the best promotional effect on the growth of vegetables.

In our experiment, the decrease in heavy metal accumulation in vegetables was mainly due to the decrease in the availability of heavy metals in soil. These results were consistent with the documented tests in which the bioavailability of heavy metals was reduced by adding an adequate quantity of either a biochar or an alkali [37,38]. The mechanisms include the specific adsorption of heavy metal ions on the surface of the additive [39], as well as precipitation and co-precipitation under a high pH condition [40]. 

The relationship between soil characteristics and soil-available Cd/Pb showed that higher soil pH, OC and CEC significantly reduced the accumulation of Cd and Pb in vegetables by decreasing the soil available Cd and Pb contents. It is well documented that adding an alkalic biochar and an industrial residue can enhance soil pH values [41]. Higher alkalinity and addition rates of the biochar and industrial residue often induce a larger reduction in soil active acidity [42]. Our results were in line with the existing data, which were clearly attributable to the alkalinity of both the added peanut shell biochar and the industrial residue. It is well known that biochar amendment with a high C content can remarkably enhance the SOC content, depending on the OC content of the added biochar and any additional quantity [43]. The high SOC content in the soil amended with both a low dosage of peanut shell biochar and alkaline mineral was probably attributable to the inhibition of biochar decomposition by the industrial residue. The details of this mechanism require further research. Biochar amendment often results in a distinct increase in CEC, depending on the biochar species used, the biochar quantity added and the soil condition [44,45]. Our results were consistent with the data in the literature. Adding a sufficient amount of peanut shell biochar with a CEC value of 17.50 cmol kg^−1^ could significantly enhance soil CEC. The CEC values were only slightly enhanced by the treatments including a mixture of 1% peanut shell biochar and alkaline mineral (B1R0.2) and alkaline mineral alone (R0.2) because of the low dosages of peanut shell biochar and alkaline mineral.

## 5. Conclusions

The following conclusions can be drawn from the results: (1) Most of the treatments in this study effectively increased the vegetable yield, and the best treatment was the mixture of 0.2% alkaline additive and a low dosage of 1% peanut shell biochar. (2) A high dosage of 9% peanut shell biochar was the best treatment for decreasing the accumulation of Cd and Pb in vegetables, but it had an inhibiting effect on the growth of chrysanthemum coronarium. The effect of the treatment with 0.2% alkaline mineral mixed with a low dosage of 1% peanut shell biochar on decreasing the accumulation of Cd and Pb in vegetables was second only to that of the treatment with 9% peanut shell biochar. (3) The reason for the reduction in Cd and Pb in vegetables was that peanut shell biochar and alkaline mineral, or their combination, could reduce the available Cd and Pb contents by increasing the soil pH and organic carbon and CEC contents. (4) There was a significant and positive interaction between the added biochar and alkaline mineral in reducing the activity of Cd and Pb. Biochar mixed with alkaline mineral had a better effect on reducing the accumulation of Cd and Pb and promoting the growth of vegetables.

## Figures and Tables

**Table 1:** The basic chemical properties of peanut shell biochar, alkaline mineral and soil (mean values ± standard deviations).

Parameters	Peanut Shell Biochar	Alkaline Mineral	Soil
pH	9.72 ± 0.65	9.87 ± 0.68	4.73 ± 0.88
Organic Carbon (%)	42.30 ± 0.32	0.01 ± 0.00	1.53 ± 0.14
CEC (cmol^+^·kg^−1^)	17.50 ± 0.14	20.13 ± 1.23	4.53 ± 0.85
Total Cd (mg·kg^−1^)	0.16 ± 0.06	n.d	5.26 ± 0.69
Total Pb (mg·kg^−1^)	5.46 ± 0.24	n.d	1110.00 ± 96.32
DTPA Cd (mg·kg^−1^)	0.06 ± 0.00	n.d	2.45 ± 0.23
DTPA Pb (mg·kg^−1^)	2.35 ± 0.05	n.d	321.70 ± 22.21
Available N (mg·kg^−1^)	211.00 ± 9.87	145.66 ± 5.67	112.83 ± 3.25
Available P (mg·kg^−1^)	317.90 ± 15.63	0.94 ± 0.16	48.90 ± 1.54
Available K (mg·kg^−1^)	33,924.00 ± 368.12	269.21 ± 24.13	54.67 ± 0.53

n.d: under detectable level.

**Table 2:** Correlation analysis between soil available Pb/Cd contents, vegetable Pb/Cd contents, vegetable biomass and soil characteristics.

Correlation Coefficient (n = 28)	Crown Daisy			Lettuce			Long-Leaf Lettuce		
	pH	CEC	SOC	pH	CEC	SOC	pH	CEC	SOC
Soil-available Cd content	−0.58 **	−0.50 **	−0.50 **	−0.78 **	−0.69 **	−0.75 **	−0.70 **	−0.33	−0.52 **
Soil-available Pb content	−0.67 **	−0.40 *	−0.51 **	−0.71 **	−0.65 **	−0.68 **	−0.80 **	−0.57 *	−0.73 **
Shoot Cd content	−0.92 **	−0.63 **	−0.55 **	−0.31	−0.73 **	−0.70 **	−0.85 **	−0.70 **	−0.50 **
Root Cd content	−0.89 **	−0.55 **	−0.45 *	−0.79 **	−0.59 **	−0.59 **	−0.82 **	−0.62 **	−0.50 **
Shoot Pb content	−0.63 **	−0.69 **	−0.48 *	−0.64 **	−0.68 **	−0.64 **	−0.55 **	−0.56 **	−0.32
Root Pb content	−0.93 **	−0.62 **	−0.54 **	−0.83 **	−0.77 **	−0.76 **	−0.77 **	−0.86 **	−0.68 **
Root biomass	0.08	−0.35	−0.33	0.77 **	0.41 *	0.41 *	0.88 **	0.31	0.45 *
Shoot biomass	0.02	−0.45 *	−0.42 *	0.81 **	0.27	0.34	0.82 **	0.18	0.39 *

Note: * and ** indicate significant correlation at the 0.05 and 0.01 levels (bilateral), respectively. Correlation coefficients of 0.8~1.0, 0.6~0.8, 0.4~0.6, 0.2~0.4 and 0.0~0.2 represent strong correlation, moderate correlation, weak correlation, very weak correlation and no correlation, respectively. A negative value indicates a negative correlation.

**Table 3:** Correlation analysis between vegetable Pb/Cd contents and soil available Pb/Cd contents.

Correlation Coefficient (n = 28)	Crown Daisy		Lettuce		Long-Leaf Lettuce	
	Soil Available Cd Content	Soil Available Pb Content	Soil Available Cd Content	Soil Available Pb Content	Soil Available Cd Content	Soil Available Pb Content
Shoot Cd/Pb content	0.52 **	0.53 **	0.59 **	0.84 **	0.62 **	0.64 **
Root Cd/Pb content	0.47 *	0.76 **	0.72 **	0.79 **	0.59 **	0.77 **

Note: * and ** indicate significant correlation at the 0.05 and 0.01 levels (bilateral), respectively. Correlation coefficients of 0.8~1.0, 0.6~0.8, 0.4~0.6, 0.2~0.4 and 0.0~0.2 represent strong correlation, moderate correlation, weak correlation, very weak correlation and no correlation, respectively. A negative value indicates a negative correlation.

## Data Availability

The datasets used or analyzed in this study are available from the corresponding author upon request.
